# Analysis of the Interaction Interfaces of the N-Terminal Domain from *Pseudomonas aeruginosa* MutL

**DOI:** 10.1371/journal.pone.0069907

**Published:** 2013-07-26

**Authors:** Virginia Miguel, Elisa M. E. Correa, Luisina De Tullio, José L. Barra, Carlos E. Argaraña, Marcos A. Villarreal

**Affiliations:** 1 Centro de Investigaciones en Química Biológica de Córdoba (CIQUIBIC), CONICET, Departamento de Química Biológica, Facultad de Ciencias Químicas, Universidad Nacional de Córdoba, Ciudad Universitaria, Córdoba, Argentina; 2 Instituto de Investigaciones en Físico-Química de Córdoba (INFIQC), CONICET, Departamento de Matemática y Física, Facultad de Ciencias Químicas, Universidad Nacional de Córdoba, Ciudad Universitaria, Córdoba, Argentina; Wake Forest University, United States of America

## Abstract

Mismatch Repair System corrects mutations arising from DNA replication that escape from DNA polymerase proofreading activity. This system consists of three main proteins, MutS-L-H, responsible for lesion recognition and repair. MutL is a member of GHKL ATPase family and its ATPase cycle has been proposed to modulate MutL activity during the repair process. *Pseudomonas aeruginosa* MutL (PaMutL) contains an N-terminal (NTD) ATPase domain connected by a linker to a C-terminal (CTD) dimerization domain that possesses metal ion-dependent endonuclease activity. With the aim to identify characteristics that allow the PaMutL NTD allosteric control of CTD endonuclease activity, we used an *in silico* and experimental approach to determine the interaction surfaces of *P. aeruginosa* NTD (PaNTD), and compared it with the well characterized *Escherichia coli* MutL NTD (EcNTD). Molecular dynamics simulations of PaNTD and EcNTD bound to or free of adenosine nucleotides showed that a significant difference exists between the behavior of the EcNTD and PaNTD dimerization interface, particularly in the ATP lid. Structure based simulations of MutL homologues with endonuclease activity were performed that allowed an insight of the dimerization interface behavior in this family of proteins. Our experimental results show that, unlike EcNTD, PaNTD is dimeric in presence of ADP. Simulations in mixed solvent allowed us to identify the PaNTD putative DNA binding patch and a putative interaction patch located opposite to the dimerization face. Structure based simulations of PaNTD dimer in presence of ADP or ATP suggest that nucleotide binding could differentially modulate PaNTD protein-protein interactions. Far western assays performed in presence of ADP or ATP are in agreement with our *in silico* analysis.

## Introduction

Mismatch Repair System (MMR) corrects mutations arising from DNA replication that escape from DNA polymerase proofreading activity, and prevents recombination between partially homologue sequences (homeologue recombination) [1]. This system has been extensively characterized in *E. coli* where three main proteins, MutS-L-H, are responsible for lesion recognition and repair. MutS recognizes mispaired bases and recruits MutL, a matchmaker protein that coordinates the action of most of the proteins involved in repair [1]. This ternary complex (DNA-MutS-L) activates MutH endonuclease, which cleaves unmethylated GATC sites transiently generated during replication, allowing strand discrimination [2]. MutL homologues from several organisms that lack MutH, including eukaryotes and most bacteria, have been found to possess a latent endonuclease activity essential for DNA strand discrimination [3]–[9]. This activity is dependent on the integrity of a metal binding motif located within MutL C-terminal domain (CTD). This motif, and therefore endonuclease activity, is absent in *E. coli* MutL [3], [4], [10].

MutL belongs to the GHKL ATPase family, which includes gyrase GyrB, Hsp90, histidine kinases and MutL [11]. All members of this family share a well conserved N-terminal domain (NTD) that contains an ATPase active site [12]. In MutL, this domain is connected by a linker to a non-conserved CTD dimerization domain [12]. Although this family lacks a conventional ATPase signature motif, it shares four conserved sequence motifs (I-IV), responsible for ATP binding [12]. ATP-binding induced conformational changes are involved in the signaling of these proteins physiological activity [11], [13]. The crystal structure of LN40, the 40 kDa NTD from *E. coli* MutL (here on denominated EcNTD), as well as human and yeast NTD MutL homologues have been determined [12]–[14]. EcNTD is made up of two α/β hemi-domains, sub-domain I (res. 1–209) and sub-domain II (res. 210–331) [12]. Both sub-domains contain a portion of the ATP catalytic site, but this is mainly made up of the sub-domain I. The first hemi-domain contains the four ATP binding motifs (I–IV) characteristic of the GHKL ATPase super-family [12]. On the other hand, the sub-domain II has a positively charged cleft capable of DNA binding, and it is suggested to have evolved from a RNA-binding domain [12]. EcNTD dimerizes upon AMPPNP binding due to the ordering of the dimerization interface which consists of four loops (L1, L2, L3 and L45) and the ATP lid [15]. L1 encompasses the first 19 N-terminal residues that contact the ATP binding site of the other subunit in the dimer [15]. L3 interacts with γ-phosphate while motif III corresponds to the ATP lid, which blocks the ATP binding site.

An interesting question is how MutL ATP binding and hydrolysis are integrated into the mismatch repair machinery. The endonuclease activity of MutL is expected to be a regulated activity, since it has to be strand-specific [5]–[7], [16]. NTD nucleotide-dependent conformational changes observed in prokaryotic and eukaryotic MutL homologues, and particularly ATP binding, have been involved in this allosteric control [5], [7], [16]. Recently, a physical NTD-CTD interaction has been demonstrated for *Aquifex aeolicus* MutL homologue, which possesses endonuclease activity [17]. Therefore, to better understand and elucidate the biochemical and structural regulatory mechanisms underlying CTD endonuclease activity, a deep understanding of the characteristics of NTD from MutL homologues that possess this activity is needed.

Although *E. coli* MMRS has been extensively studied, little is known about this system in the gram negative bacteria *Pseudomonas aeruginosa,* an opportunistic pathogen that affects inmuno-compromised and Cystic fibrosis patients [18]. *P. aeruginosa* MRS lacks MutH, and recently, the endonuclease activity of CTD of *P. aeruginosa* MutL has been described [9]. Addition of ATP inhibits PaMutL nicking activity suggesting a regulatory role of adenine nucleotide binding [9]. In this work, we have focused on the characterization of *P. aeruginosa* NTD (PaNTD) with the aim to characterize its structure and dynamics and to help the understanding of the allosteric control of NTD on the endonuclease activity of CTD. We used an *in vitro* and *in silico* approach to determine the effect of nucleotide binding in PaNTD structure and dynamics and to characterize its interaction surfaces. Size exclusion chromatography assays show that unlike EcNTD, PaNTD is dimeric in presence of ADP. Molecular dynamics simulations of PaNTD models and EcNTD crystal structures showed that a significant difference exists in the behavior of the EcNTD and PaNTD dimerization interface explaining the behavior observed *in vitro*. Mixed solvent and structured based model simulations of PaNTD allowed us to identify and characterize the PaNTD DNA binding patch and a potential protein-protein interaction site. These simulations suggest that nucleotide binding could differentially modulate PaNTD protein-protein interactions.

Our in silico results give theoretical support and are in agreement with experimental results. The implications of these PaNTD characteristics in the regulation of MutL activity are discussed.

## Materials and Methods

### Bacterial Strains, Plasmids, and Chemicals

*E. coli* Bl21 (λDE3) and expression plasmid pET-15b were obtained from Novagen. *E. coli* XL1-Blue was supplied by Stratagene. The pGEM-T Easy cloning vector and DNA modification enzymes were obtained from Promega. The expression vector pTYB12 and Chitin column were purchased from New England Biolabs. His-binding resin was obtained from Invitrogen. BSA used as molecular weight standards and for western blot analysis was supplied by Sigma. Bradford reagent was obtained from Bio-Rad.

### Cloning of *E. coli* and *P. aeruginosa* MutL N-terminal and *P. aeruginosa* C-terminal Domains

The *P. aeruginosa mutL* gene was amplified from genomic DNA by PCR using primers MLPgS (5′-ATCATATGAGTGAAGCACCGCGTATCC-3′, NdeI site underlined) and MLPgA (5′-ATGGATCCTCTTGGACAAAGCGCATA-3′, BamHI site underlined). The amplified PCR fragment was cloned into pGEM-T Easy cloning vector to generate plasmid pG-PaMutL. The NdeI–EcoRI fragment from plasmid pG-PaMutL was then cloned in the expression vector pET-15b to generate plasmid Pet-PaMutL. The *P. aeruginosa* MutL-NTD sequence (PaNTD, amino acids 1- 339) was amplified from pET-PaMutL by PCR with primers MLPgS and primer Cris-cLpA (5′-GGAATTCAGTCGTCGGGACGGACCTCGC-3′, EcoRI site underlined). The *E. coli* MutL-NTD sequence (EcNTD, amino acids 1- 342) was amplified from pET-15b-coliMutL provided by Feng [19] by PCR with primers NCcrisS (5′-GCCATATGCCAATTCAGGTCTTACCGC-3′, NdeI site underlined) and the primer NCcrisA (5′-CGAATTCAATCGTCCAGCGGTAGCGGCG-3′, EcoRI site underlined). The PCR product was cloned into pGEM-T Easy and then inserted into the NdeI-EcoRI restriction sites of pET-15b vector for expression and further purification.

For expression of PaCTD, a plasmid carrying a MutL derivative devoid of most of the N-terminal ATP binding region (pTYB12-PaMutLΔ1–224) was used [9].

### Protein Purification

PaCTD was purified as described [9]. As a result, purified free-of-tag PaCTD was obtained. for PaNTD and EcNTD, *E. coli* strain BL21 (λDE3) transformed with pET-PaNTD or pET-EcNTD respectively, were grown at 37°C in Luria–Bertani (LB) medium containing 200 µg/ml ampicillin and 0.5% glucose to an absorbance at 600 nm of 0.6. Subsequently, IPTG was added to a final concentration of 1 mM, and the cells were incubated at 37°C for 1 h. Cells were harvested by centrifugation and suspended in 20 mM HEPES (pH 7.4), 0.5 M NaCl and 13.3% (v/v) glycerol. Cell suspension was processed with EmulsiFlex-C3 homogenizer and centrifuged at 100000 g for 30 min. Soluble fractions were incubated ON with His-Bind resin. Protein was eluted from the column with elution buffer [20 mM HEPES (pH7.4), 0.5 M NaCl and 13.3% (v/v) glycerol and 0.2 M imidazole]. Proteins were obtained with a purity >95%. Immediately after column elution, buffer was exchange using a YM-10 centricon for Protein Buffer [20 mM HEPES pH:7.4; 150 mM KCl; 10% glycerol (v/v) and 1 mM DTT]. Protein concentration was determined by Bradford assay using BSA as a standard and aliquots were stored at −70°C.

### Determination of NTD Oligomeric State

The oligomeric state of purified PaNTD and EcNTD in the apo form or bound to ADP or ATP, were determined by gel filtration chromatography in a Superose 12 10/30 columns (Amersham Pharmacia Biotech) equilibrated with 20 mM Tris–HCl, pH 7.9; 150 mM KCl; 5 mM MgCl_2_; 1 mM DTT. 1 mg/ml PaNTD and 2 mg/ml EcNTD incubated in absence or in presence of ADP or ATP were applied to the column, elution was carried out at room temperature at a flow rate of 0.5 ml/min and the absorbance was measured at 280 nm. Column calibration was performed using BSA of 45 and 66 kDa as molecular weight standards.

EcNTD chemical crosslinking were performed as described [20]. EcNTD in 20 mM HEPES (pH: 7.4), 150 mM KCl, 10% glycerol, 5 mM MgCl2 and 1 mM DTT was incubated with 1 mM EDTA, ADP, ATP or AMPPNP at room temperature for 1 h and then 4°C ON. Protein DSS chemical cross-linking was performed at 4°C for 1 h and samples were analyzed on a 10% SDS–PAGE.

For native PAGE, samples were loaded onto 10% polyacrylamide/bys-acryilamide (30%/0.8%) gels and ran in 25 mM Tris pH 7.6, 200 mM Glycine buffer at 4°C.

### Far Western Analysis

To perform far western assays, His6-PaNTD (20 pmol) as a positive control, BSA (20 pmol) as negative control and purified non-tag PaCTD (6.5 pmol) were spotted onto Protran nitrocellulose membranes (0.2 µm, BioSciences). The membranes were blocked for 1 h at room temperature in blocking buffer 20 mM Tris–HCl, pH 8.0, 0.15 M KCl, 1 mM EDTA (Buffer B) supplemented with 0.1% Triton X-100 and 5% milk, and then incubated with 0.6 µM of His6-PaNTD in Buffer B supplemented with 5 mM MgCl_2_ (Buffer C), Buffer C with ADP 0.1 mM or Buffer C with ATP 0.1 mM overnight at 4°C. After washing, the membranes were incubated with rabbit anti-His6 antibody (1/20,000, Santa Cruz Biotechnology) for 3 h at room temperature, washed, and then incubated for 1 h with IRDye 800CW-conjugated goat anti-rabbit antibody (LI-COR Bioscience). The data were visualized using an Odyssey infrared imaging (LI-COR Bioscience) instrument. Spots were quantified using the software ImageJ [21].

### NTD Homology Modeling

A homology model of PaNTD was made using MODELLER 9v8 [22]. Crystal structures of EcNTD bound to different nucleotides were used as a template (PDB accession numbers: 1B62; 1B63; 1NHH; 1NHI and 1NHJ). The sequences were aligned using the ClustalW software [23]. Models were built with automodel class, the model with the lowest value of the MODELLER objective function [22] was picked and model quality was assessed using QMEAN (score = 0.6) [24]. Since there are some regions missing in the crystal structure of apo EcNTD, the nucleotide bound structures were used to generate a unique model of PaNTD that was used, bound or unbound to ATP, to perform simulations of holo and apo PaNTD, respectively. Due to the templates used, the model created would correspond to a nucleotide bound conformation of PaNTD. For the simulations in the holo states the nucleotide was docked, while for the simulations in the apo state, no ligand was added.

WebMod server [25] was used to model BsNTD and TmNTD 3D structures used for structure based models simulations, since these proteins tertiary structures have not been determined experimentally. BsNTD model was constructed using EcNTD template (PDB:1B63A). These two proteins have an identity of 41%. For TmNTD a model was constructed using EcNTD template (PDB:1B63A). These two proteins have an identity of 36%.

### Molecular Dynamics in Water

Molecular dynamics (MD) simulations in explicit water were carried out for 200 ns for (i) the apo form of PaNTD, (ii) PaNTD complexed with ATP, and (iii) *E. coli* MutL NTD (EcNTD) in the apo form starting from the crystal structure of ECNTD [12] (PDB entry: 1B63), where the AMPPNP bound to this structure was removed (iv) holo EcNTD using the same structure, where AMPPNP was replaced with ATP.

The amino acid side chains were charged according to the pKa of the amino acid in the 3D structure calculated with PROPKA [26] and assuming a pH of 7 for the buffer. The total charge on the apo proteins were +12 and +7 for PaNTD and EcNTD, respectively. In all cases we added the necessary amount of Cl– ions to obtain an electrically neutral system plus excess of Na+ and Cl– to reach a final concentration of 150 mM NaCl. The proteins were solvated with between 1.5×10^4^ and 2×10^4^ water molecules. The GROMOS96 53a6 [27] force field with modifications in the torsional potential of the backbone (Villarreal MA and Leiva EPM., unpublished results) was employed for the protein, and the SPC/E model for the water [28]. The bonds in the peptide were constrained using the LINCS algorithm [29], while the water molecules were kept rigid using SETTLE [30]. The time step for the integration of the equation of motions was 5 fs due to the use of virtual sites and mass repartitioning [31]. The electrostatic interactions were handled with the SPME version of the Ewald sums [32], with a real space cutoff of 0.9 nm, a grid spacing of 0.12 nm, and a cubic interpolation. The van der Waals interactions were cut-off at 1.4 nm. The temperature was maintained at 300K by separately coupling the protein and the water using the velocity rescale algorithm of Bussi et. al [33], which ensure a proper canonical ensemble. The system pressure was coupled isotropically to a reference pressure of 1 bar with a relaxation constant of 2.0 ps, using the Berendsen algorithm [34].

Three minimization steeps were carried out successively restraining the protein, the backbone and finally the Cα with steepest descendent. Then, the protein was allowed to equilibrate at 150K and 300K for 50 ps. In the calculation of the root mean square deviation (RMSD) and radius of gyration, the first 12 and 9 residues of PaNTD and EcNTD, respectively, were not taken into account because of their intrinsic flexibility. Cluster analysis was used to identify the representative structures of apo and holo-protein. The linkage method was used, were a structure is added to a cluster when its distance to any element of the cluster is less than a cut-off, which in this case was 0.2 nm. The secondary structure content was evaluated with the DSSP algorithm [35].

### Structure Based Simulations

Given the high disorder and mobility of the N- and C-terminal residues observed in the MD simulations in explicit solvent, the first and last five residues were excluded in these simulations. All-atom structured-based models (SBM) for the monomeric systems were prepared with SMOG@ctbp server (http://smog.ucsd.edu/) [36]. The initial structures used for these constructions were the same used for all-atom MD. The simulation protocol was the same as described in [37]. The temperature was set to 0.90 Tf of apo EcNTD, were Tf is the folding temperature of the model determined in trial runs. We also performed SBM simulations of PaNTD dimers bound to ADP or ATP. A model of PaNTD dimer was constructed using the crystal structures of *E. coli* MutL NTD bound to different nucleotides as templates and the temperature was set to 0.95 Tf.

All the SBM simulations were extended until the root mean square fluctuations (RMSF) calculated with the first and second half of the trajectories were identical. This usually required 10^7^ integration steps.

### Molecular Dynamics in Mixed Solvent (H_2_O/iPrOH)

The central structure of the main cluster found in the MD simulations of the apo and holo PaNTD in water was used to perform a MD simulation in mixed solvent as described before [38]. The simulated systems consisted in a protein plus ∼13×10^3^ water and ∼0.5×10^3^ isopropyl (iPrOH) molecules, which correspond to a 20% solution. Each system was minimized, equilibrated, and simulated as described above. Simulations were carried out for 50 ns. Spatial distribution function (SDF) was calculated using a bin of 2.5 Å. VMD [39] was used for rendering, using an isosurface representation and a density isovalue of 20.

All simulations and analysis were performed using the GROMACS 4.0.7 simulation package (http://www.gromacs.org) [37]. VMD 1.8.7 [36] (http://www.ks.uiuc.edu/Research/vmd/) was used for visualization and figure rendering, and XMGRACE (http://plasma-gate.weizmann.ac.il/Grace/) was used for figure plotting.

## Results

### Molecular Dynamics Simulations show a Differential Effect of ATP Binding on *E. coli* and *P. aeruginosa* MutL ATP Lid Dynamics

We aim to determine if a differential behavior exists between N-terminal domains (NTD) of a MutL homologue that possesses with one that lacks of endonuclease activity. We hypothesized that NTD could behave differentially since the former have to cope with an additional activity. As mentioned before, ATP binding has been involved in the allosteric control of CTD activity [5], [7], [17], [40]. Taking this into account, we performed all-atom molecular dynamics (MD) simulations of monomeric ATP-bound or ATP-free PaNTD and *E. coli* LN40 (here on denominated EcNTD).

**Figure 1:**
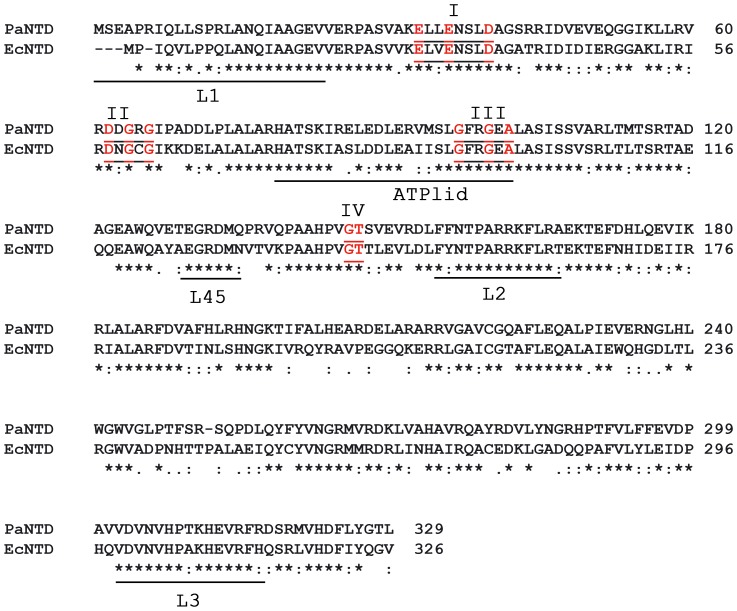
Sequence alignment of MutL PaNTD and EcNTD. ATP binding motifs conserved among GHKL ATPase superfamily (I–IV) are indicated. Red residues correspond to conserved residues within motifs. Loops involved in NTD dimerization (L1, L2, L3 and L45), as well as ATP lid, are indicated with horizontal bars. Amino acids that are identical (*), strongly similar (**:**) or weakly similar (**.**) are indicated.

PaNTD amino acid sequence has an identity of 64% with EcNTD, and a similarity of 79% (Figure 1). Due to the lack of a crystal structure for PaNTD, a homology model was made using MODELLER [22] with EcNTD crystal structures bound to different nucleotides as templates (see Matherial and Methods). The PaNTD model can be superimposed with the crystal structure of EcNTD bound to AMPPNP (PDB: 1B63) with a RMSD value of 0.2 nm. Residues in the ATP binding site of EcNTD that directly bind the nucleotide are well conserved in PaNTD (Figure 1). Also, the four sequence motifs (I–IV) involved in nucleotide binding that are characteristic of GHKL ATPase family can be identified in the PaNTD sequence (Figure 1). According to sequence and structure alignment, putative PaNTD dimerization interface consists of **L1** (residues 6–24, EcNTD r. 2–20. 79% identity), **L2** (r. 154–166, EcNTD r. 150–162. 85% identity), **L3** (r. 302–316, EcNTD 299–313. 86% identity), **L45** (r. 130–135, EcNTD 126–131. 86% identity) and **ATP lid** (r. 78–101, EcNTD 74–97. 75% identity).

MD simulations in explicit water were carried out for (i) the nucleotide free (apo) form of PaNTD, (ii) PaNTD complexed with ATP (holo) (iii) EcNTD in the apo form and (iv) holo EcNTD. All simulations were run for 200 ns. Given that the model for PaNTD is based on the holo form of EcNTD which is dimeric, and that the simulations of the apo state were generated by simple removal of the ligand, the simulations of the four systems started with a very similar global conformation. During the simulations we expected to observe deviations from the initial structures for two reasons. First, it has been suggested [15] that the final conformation of the protein is only reached when the protein dimerizes and the monomeric form may deviate from the initial structure. Second, when EcNTD is crystallized without ligand, the regions which comprise the dimerization interface cannot be defined in the X-ray experiment probably due to an increased mobility of this zone [12]. The root mean square deviation (RMSD) of the Cα was calculated relative to the starting structures to assess the stability of the systems. When the first 12 and 9 residues of PaNTD and EcNTD respectively were not taken into account (given the high mobility of these regions), both proteins showed a similar behavior, reaching values between 0.30 and 0.35 nm after 200 ns (Figure S1A). These values over 0.30 nm indicate that the proteins suffer some (minor) global conformational change during the simulations and are in line with the idea that the crystal structure is achieved only in the dimeric state. Taking into account that the starting model of PaNTD has a RMSD of 0.2 nm from the EcNTD initial structure, the similar behavior for both proteins observed in Figure S1A supports the validity of the model created for PaNTD. A noticeable jump in the RMSD curve of apo EcNTD is observed between 5 and 25 ns, where it reaches values near 0.4 nm (Figure S1A). After that time, the RMSD curve returns to values near 0.3 nm. This jump would indicate that the apo EcNTD can explore conformations which are not readily available to either the holo EcNTD or the PaNTD in both states (apo and holo).

The difference in total secondary structure content between the holo and apo forms of both proteins is shown in Figure S1B. These curves consistently decrease as a function of time, which indicates that for both proteins the apo form has less secondary structure than the holo form. Between both proteins there were no noticeable differences. This decrease in secondary structure when the ATP is removed from both proteins could be related to the aforementioned [12].

**Figure 2:**
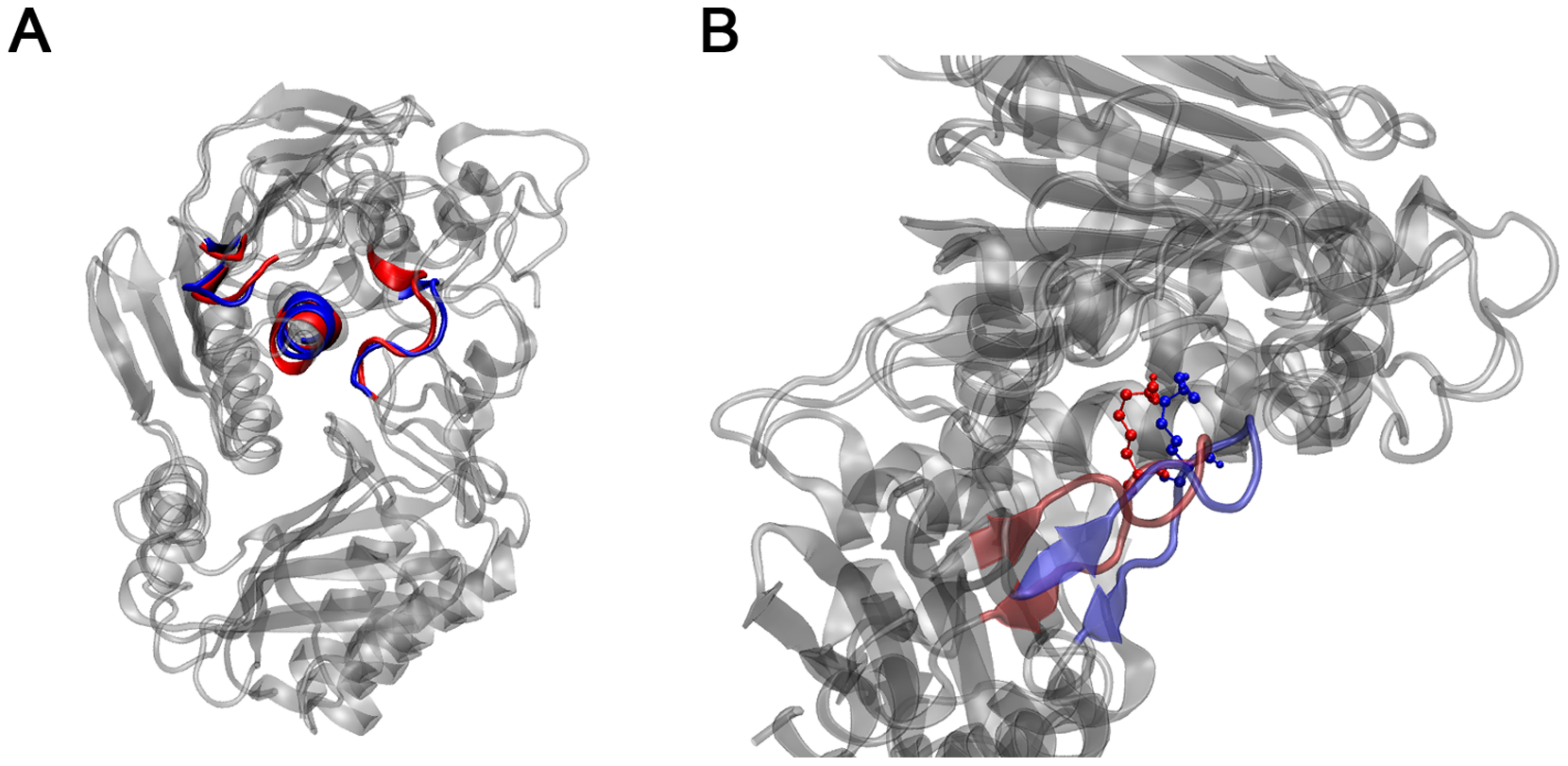
Cluster analysis of MutL PaNTD and EcNTD. A) The central structure of the main cluster of ATP-bound PaNTD and EcNTD were compared. The corresponding ATP binding motives (I–IV) are colored (PaNTD in red; EcNTD in blue). B) Relative position in the superimposed structures mentioned of the conserved residue lysine of motif V (K307 in EcNTD, blue and 310 in PaNTD, red).

In order to compare the four protein systems, a cluster analysis was carried out for each protein using the 200 ns and a cut-off of 0.2 nm. The central structure from the main cluster of the ATP-bound PaNTD and EcNTD were determined. These structures can be superimposed using the Cα of their respective ATP binding motifs (I–IV) with a RMSD value of 0.15 nm (Figure 2A). This measure of the local similarity of PaNTD model indicates a rather small divergence in the position of residues responsible for ATP hydrolysis between these two structures. Also, the conserved residue lysine of motif V (K307 in EcNTD and 310 in PaNTD) maintains its relative position in both proteins (Figure 2B). This residue inserts into the active site where it contacts the γ-phosphate and is key for the correct work of the enzyme [15]. The central structure from the main cluster of apo EcNTD and the one corresponding to the cluster that appears during the first 25 ns were compared in order to determine the main changes that are produced in this protein structure and are responsible for the jump observed in the RMSD curve in Figure S1A. These structures can be superimposed with an RMSD of 0.4 nm, with noticeable differences in the dimerization interface, principally in the conformation of the ATP lid (data not shown).

**Figure 3:**
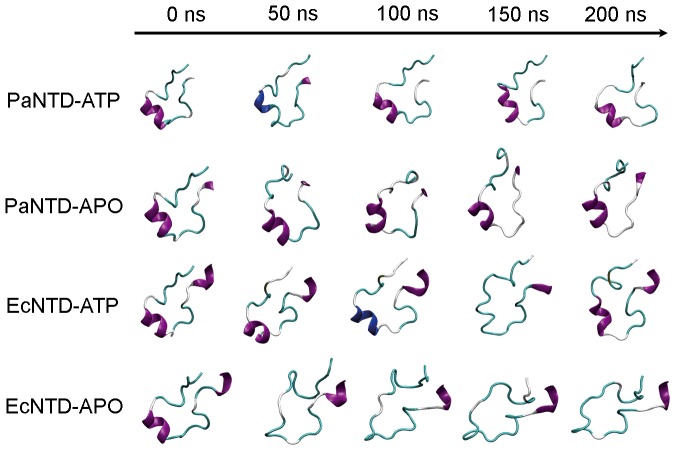
Time evolution of ATP lid secondary structure in all atom MD. Secondary structure of the ATP lid from the different systems was analyzed. The secondary structure information was obtained using the do_dssp program (also see Figure S2) and representative structures were taken from the MD. Purple: alpha helix; blue: 3-helix; cyan: turn; white: coil.

The RMSD is a global measure of the similarity between structures, and some important but more subtle structural changes could be masked by values of around 0.3 nm. The same masking effect could be taking place when analyzing the total secondary structure. A careful analysis of the trajectories showed a distinct behavior of the ATP lid between PaNTD and EcNTD. Snapshots of the conformation sampled by the ATP lid (residues 74–97 for EcNTD and 78–101 for PaNTD) along the trajectories are shown in Figure 3. Figure S2 shows the secondary structure of the full dimerization interface as a function of time. Apo EcNTD ATP lid undergoes a significant structural change early in the simulation. The helix between residues 81–88 is lost and not recovered for the rest of the simulation (Figure 3 and Figure S2). On the other hand, in the holo form of EcNTD as well in both forms of PaNTD, the ATP lid maintains its α-helical structure along the whole MD trajectory (Figure 3 and Figure S1). Ban & Yang experimental results [15] show that in EcNTD crystal structure, ATP lid becomes more mobile in ADP-bound and nucleotide free forms in comparison to ATP-bound, but remains partially structured. Although EcNTD–ADP crystal structure is dimeric, probably due to crystal packing, this complex is a monomer in solution. Also, in the absence of the γ-phosphate, ATP lid becomes more mobile as indicated by higher B values [15].

An analysis of PaNTD and EcNTD ATP lid alignment shows that ATP lid helix is conserved (Figure 1). Nevertheless when the highly reliable and experimentally calibrated AGADIR prediction algorithm [41] was used to calculate the α-helix propensity of these two isolated regions, the alpha helix propensity was larger for the PaNTD sequence than for the EcNTD (Figure S3). This result is in line with MD simulations.

### Structure Based Molecular Dynamics Simulations in Presence or in Absence of Adenine Nucleotides Reflect a Differential Behavior of PaNTD and EcNTD Dimerization Interface

Based on the funneled nature of the energy landscape of protein folding [42], structure based models (SBM) provide a computationally efficient and reliable model to explore the large scale molecular motions of proteins which are no reachable with more detailed models as used in the previous section.

**Figure 4:**
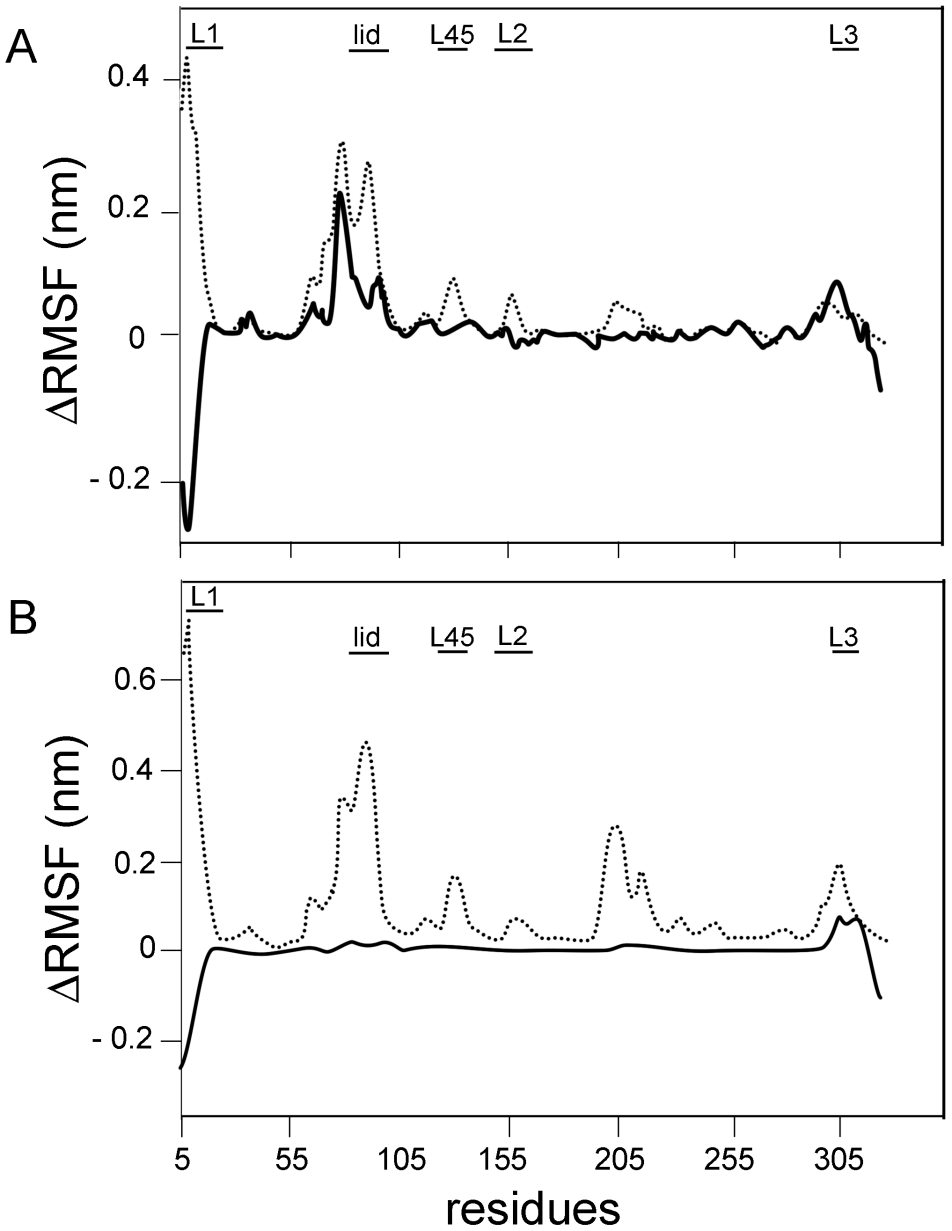
Analysis of PaNTD and EcNTD monomers residues mobility using structure based models (SBM). Root Mean Squeare Fluctuation (RMSF) difference between A) apo and ATP-bound monomer and B) ADP and ATP-bound monomer, for EcNTD (dotted line) and PaNTD (full black line) were calculated from SBM MD simulations. Dimerization interface (L1, L2, L3, L45 and ATP lid) is indicated with black horizontal bars.

MD simulations of SBM were performed to further analyze the difference between proteins under study. These simulations were run for a time long enough to guarantee that the root mean squared fluctuations (RMSF) calculated with one half of the trajectory is identical to the calculated with the second half. This kind of converged picture is not possible to achieve with the 200 ns of simulations that were performed with the most complete model used in the previous section, and it is of fundamental importance as we seek for the difference between two of such RMSF curves. Figure 4A shows the difference in RMSF (Δ RMSF) between the apo and ATP bound forms of each protein (full line: PaNTD; dotted line: EcNTD). It is observed that the apo forms showed a more flexible structure for both species. This result is in line with the reduction in secondary structure observed for apo proteins during the explicit solvent MD simulations (Figure S1B). More importantly for this work, this analysis clearly revealed the presence of 5 regions with different behavior between EcNTD and PaNTD. Residues corresponding to the dimerization interface, namely loops L1, L2, L3, L45 and the ATP lid were more mobile in apo EcNTD than in apo PaNTD. Also, the difference in RMSF between the ADP and ATP bound forms was calculated (Figure 4B). When ΔRMSF for ADP and ATP-Bound EcNTD was calculated (Figure 4A, full line), it was clear that the ADP bound showed higher mobility of the dimerization interface. This is reflected as high positive ΔRMSF values. On the other hand, ΔRMSF of PaNTD with ATP or ADP bound states where quite similar (Figure 4B, dotted line). This is reflected as near zero ΔRMSF values, and indicates that these ADP and ATP-bound PaNTD behave similarly.

**Figure 5:**
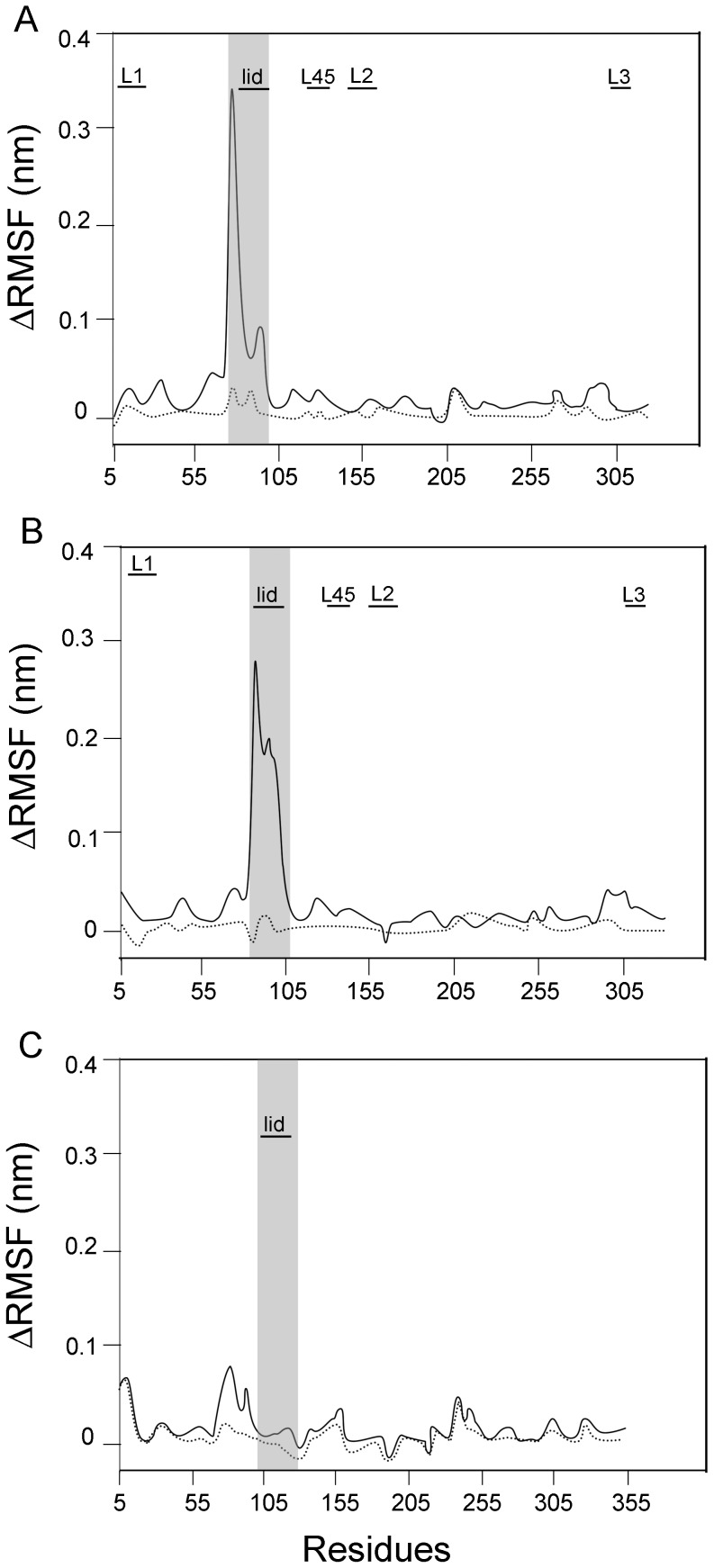
Analysis of BsNTD, TmNTD and PMS1 monomers residues mobility using structure based models (SBM). Root Mean Squeare Fluctuation (RMSF) difference between apo and ATP-bound (full black line) and ADP and ATP-bound monomer (dotted line) for; A) BsNTD B) TmNTD and C) yeast PMS1 were calculated from SBM MD simulations. Dimerization interface is indicated with black horizontal bars. ATP lid residues are marked with grey shadow.

Additionally, MD simulations of SBM were performed to analyze differences between NTD from MutL homologues with/without endonuclease activity (Figure 5). The N-terminal domains of MutL homologues with endonuclease activity from *Bacillus subtillis* (BsNTD), *Termotoga maritima* (TmNTD) and PMS1 from *Saccharomyces cerevisiae* (yNTD) were analyzed. WebMod server [25] was used to model BsNTD and TmNTD 3D structure, since these proteins tertiary structures have not been determined experimentally. SBM MD simulations were carried out for the four proteins in the nucleotide free (apo) form or bound either to ADP or ATP. Differences in RMSF (ΔRMSF) between (i) the apo and ATP bound forms or (ii) ADP and ATP of each protein were determined (Figure 5). For BsNTD a model was constructed using EcNTD template (PDB:1B63A). BsNTD (Figure 5A) display a very similar behavior as the one observed for PaNTD (Figure 4). This indicates that dimerization interface is less mobile for ADP and ATP bound BsNTD than ADP and ATP bound EcNTD. As for PaNTD, the region that possesses a similar Apo and ATP bound ΔRMSF with EcNTD corresponds to the ATP lid stem (residues ∼75–80), but for the ATP lid α-helix a reduction in ΔRMSF can be observed for both PaNTD and BsNTD (residues 80–90). For TmNTD a model was constructed using EcNTD template (PDB:1B63A). It is observed that the apo form showed a more flexible structure than the ATP-bound form (Figure 5B). No differences were observed in rmsf values between ADP and ATP-bound TmNTD (Figure 5B). The crystal structure of yeast PMS1 NTD bound to AMPPNP (PDB: 3H4LB) was used as an initial model. PMS1 apo form presented high RMSF values, particularly in the ATP lid region, where these values were 3 times larger than apo EcNTD (data not shown). More interesting, practically no differences in RMSF vales were shown when apo, ATP and ADP-bound form were compared (Figure 5C).

### *In vitro* Assays showed that PaNTD is Dimeric when Bound to ADP

Since MD simulations results indicated that there is a differential behavior of the dimerization interface of EcNTD and PaNTD, we analyzed the effect of such behavior on their oligomeric state. Ban & Yang (1998) have shown by size-exclusion chromatography that binding of ADPnP induces a transition of EcNTD from monomer to dimer [12]. EcNTD dimerization is specific of ATP binding since no detectable changes are shown with ADP [15].

**Figure 6:**
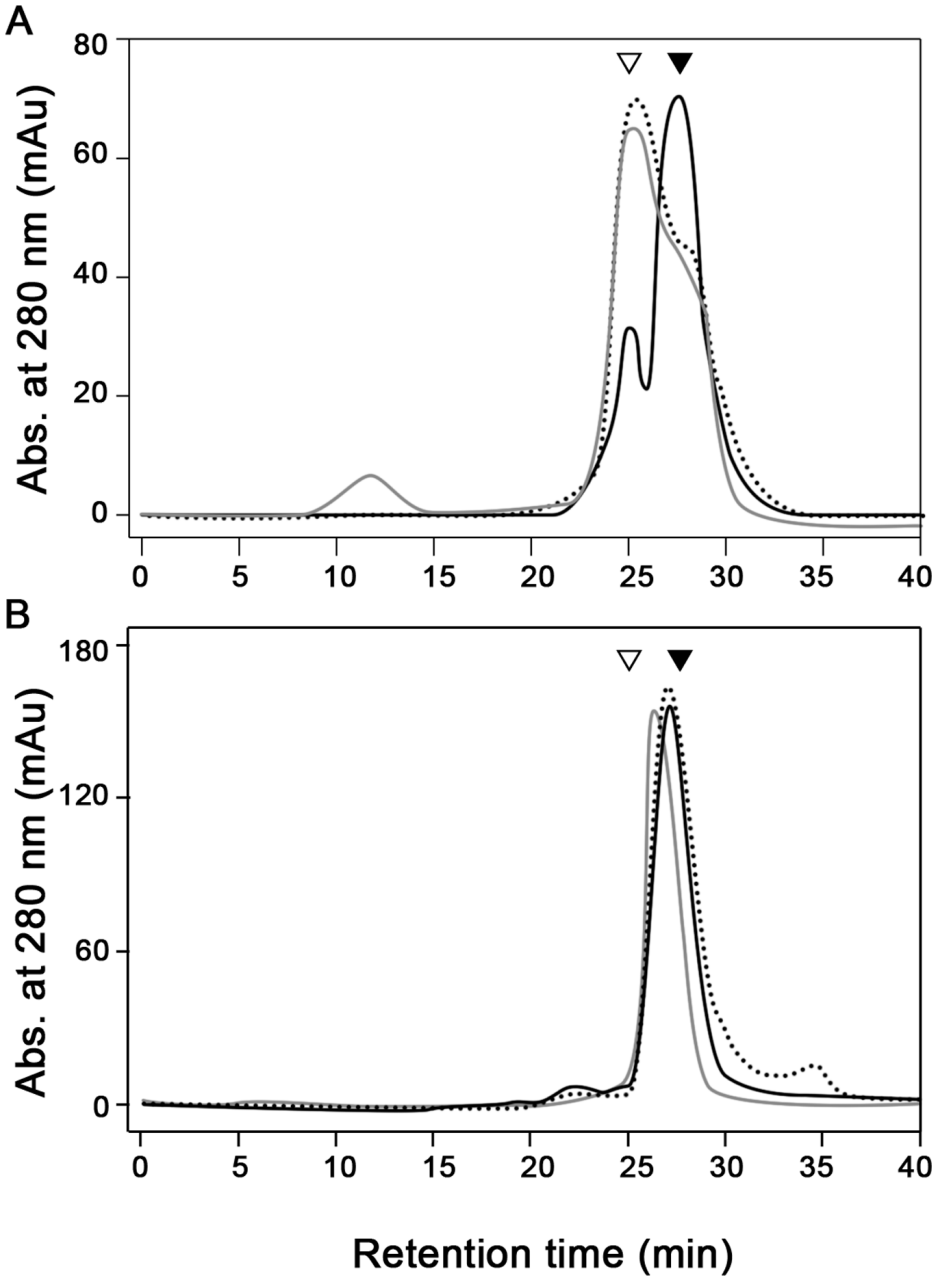
Determination of PaNTD oligomeric state using gel filtration chromatography. A typical elution profile is shown: (A) PaNTD and (B) EcNTD proteins (22 µM) nucleotide free (full black line), bound to ADP (dotted line) or bound to ATP (full grey line) were analyzed on a Superose 12 column as described in Matherial and Methods. Arrow heads indicate the elution positions of MW standards (∇ BSA 66 kDa; ▾BSA 45 kDa).

We cloned and purified recombinant PaNTD (residues 1–339 of PaMutL, monomeric MW = 37.7 kDa). When analyzed in SDS-PAGE, PaNTD presented a MW ∼40 kDa (data not shown). The oligomerization state of the apo protein was determined by gel filtration chromatography (Figure 6A). The elution profile of PaNTD presented two peaks, one corresponding to a MW of ∼40 kDa similar to that expected for the monomeric protein (MW:38 kDa) and a smaller one with an estimated molecular weight of ∼70 kDa (Figure 6A, full black line). The peak relationship dimer:monomer was aprox. 0.2∶0.8. The dimeric form of PaNTD is resistant to ON EDTA incubations as determined by native gels (Figure S4). PaNTD binding to ADP displaced monomer-dimer equilibrium as judged by size-exclusion chromatography (Figure 6A, dotted line). When incubated with ADP, the peak ratio inverted, and the dimeric form became the major species (Figure 6A). This result shows that, unlike EcNTD, PaNTD is capable of forming dimers in presence of ADP. No major differences were observed among the elution pattern of ADP or ATP bound PaNTD (Figure 6A). As a control, we cloned and purified his-tag EcNTD (residues 1- 342 of EcMutL, monomeric MW = 38.3 kDa) and the oligomerization state of the apo protein was determined by gel filtration chromatography (Figure 6B). The elution profiles of EcNTD in the apo form or bounded to ATP or ATP presented only one peak, corresponding to the monomeric protein (∼40 kDa). Since EcNTD dimerization in presence of ATP is not evidenced in size exclusion chromatography, we performed cross-linking assays (Figure S5). EcNTD was incubated ON with EDTA, ADP, ATP and AMPPNP, and EcNTD dimers were only observed in presence of AMPPNP (Figure S5), as established [15].

These results show that, unlike EcNTD, PaNTD is capable of forming dimers in presence of ADP.

### Mix-solvent MD Simulations and in vitro Assays Allowed the Detection of PaNTD Protein-protein and Protein-DNA Interaction Sites

**Figure 7:**
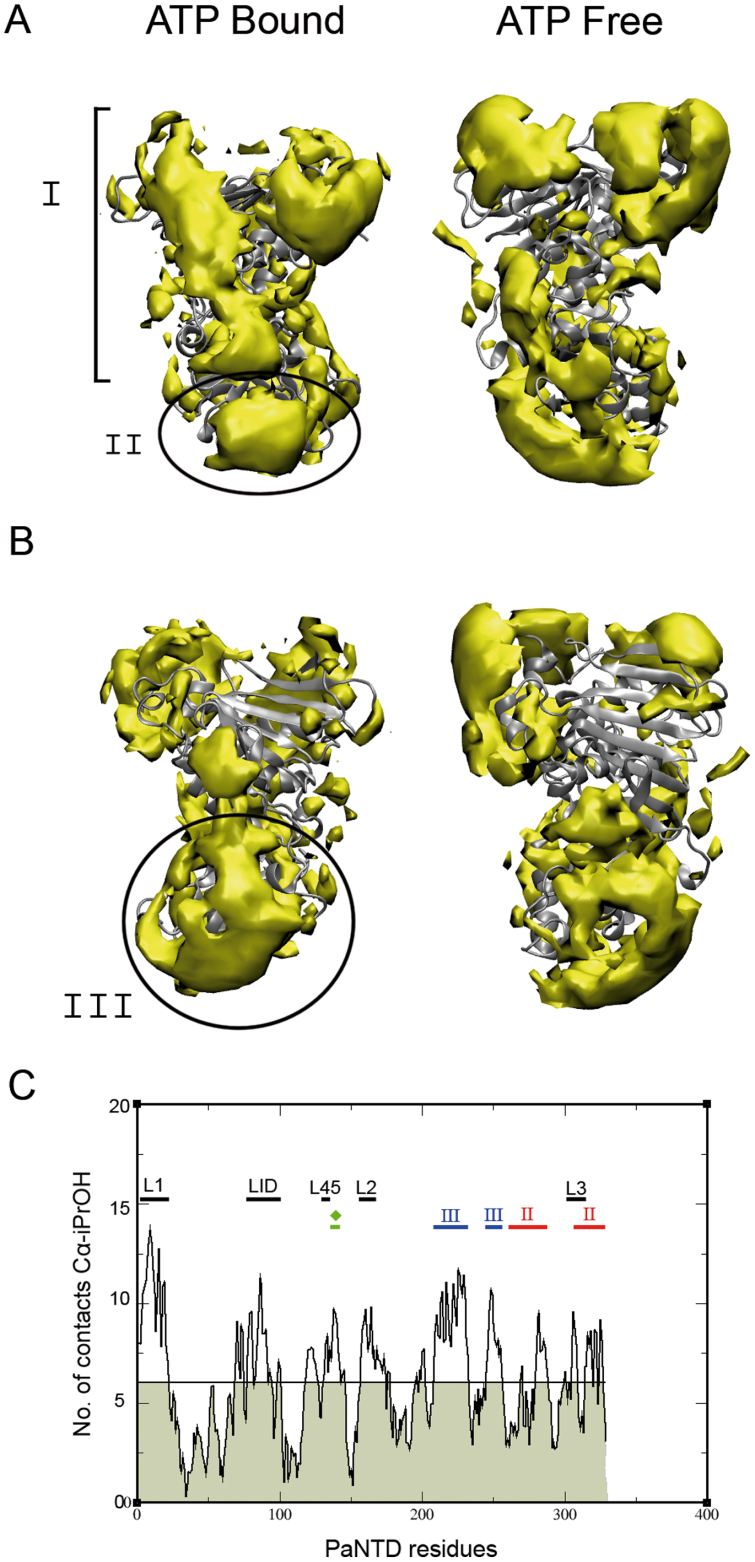
PaNTD mixed solvent MD analysis. Three-dimensional density distribution of iPrOH in ATP bound and unbound PaNTD is showed from a frontal A), and backside B) view. I: Dimerization interface; II: DNA binding patch; III: putative interaction surface. Figure rendering was made using VMD, with an isosurface representation and density isovalue of 20. C) Quantification of protein-iPrOH contacts between protein Cα and all iPrOH C1 was made using g_mindist with a cut-off of 0.15 nm. Cα contacts with iPrOH atom C1 along the MD were calculated. Dimerization interface (L1–45 and ATP lid) as determined with sequence alignment is indicated with black horizontal bars. Residues predicted to bind DNA (residues 259–276 and 307–330) (* and red bars) using the DNAbinR server and *E. coli* MutS-MutL interface mapped by Winkler et al. [45] are indicated (EcNTD r. 131–135; PaNTD r. 135–138) (• and green bar). Finally, a putative interaction interface (residues 209–230 and 245–252) (# and blue bars) was determined as high iPrOH density area. The calculated mean contact for all residues is indicates with a black line across the chart and the areas beneath this threshold were shadowed with light grey to facilitate the identification of high iPrOH density residues.

Next, we aimed to determine the possible protein-protein and protein-DNA interaction sites that could mediate PaMutL activity regulation. The central structure of the main cluster for the apo and holo PaNTD obtained in the pure water simulations were used to perform a MD simulation in a solution of water with 20% isopropyl alcohol (iPrOH) [38]. The idea behind these simulations is that regions with an increased concentration in iPrOH predict regions of the protein that are more easily desolvated, indicating putative hot spots for interactions with other molecules. This method has recently been used to determine the interaction interface of a phospholipase with lipid membranes [43]. Spatial distribution functions (SDF) were calculated for PaNTD in both states (Figure 7A–B). Also, the quantification of contacts along the MD between ATP-bound PaNTD Cα and iPrOH was included (Figure 7C). These analyses allowed the determination of PaNTD sites that preferentially bind iPrOH. Three regions that are highly enriched in iPrOH where spotted up and, therefore, considered as putative interaction interfaces. The first region is analogue to the known dimerization interface described for EcNTD (Figure 7A–I and 7C), which is formed around a hydrophobic core assembled between L1 of a monomer and the ATP lid [15]. The second region is expected to be the DNA binding patch (268–280 and 317–329), since it corresponds with the homologue region described for ECNTD [15] (Figure 7A–II and 7C). In addition, the server DNABindR [44] for prediction of protein-DNA interaction sites was used to predict PaNTD residues involved in DNA contact (Figure S6). This server is trained to predict whether a given amino acid residue is a DNA-binding residue based on its identity and the identities of its sequence neighbors [44]. EcNTD prediction was used as a control of the accuracy of the predictor. EcNTD residues predicted to be involved in DNA contact by DNABindR are in agreement with the ones described by Ban&Yang (1999) to be part of the EcMutL DNA-binding groove. Residues predicted by DNAbindR to be involved in PaNTD DNA binding are in agreement with the ones spotted out in mixed-solvent MD (Figure 7C).

The last putative interface detected is located opposite to the DNA binding patch, comprise residues 209–230 and 245–252, and currently has no assigned function (Figure 7B-III and 7C). Also, MutS-MutL interaction site detected in EcMutL by Winkler et al. [45] can be identified in the homologue PaNTD residues, as a high iPrOH density area (Figure 7C).

Finally, a comparison of iPrOH density around the apo and ATP bound protein allowed us to identify the partial loss of two PaNTD iPrOH binding sites in the nucleotide free form. The first reduction is observed in the ATP lid, and the second is located around L3 (Figure 7A). Also, there is a small reduction of iPrOH binding in the putative DNA interaction site (Figure 7A) which could translate into a loss of the NTD-DNA interaction surface.

### A Differential Effect of ADP or ATP Binding on PaNTD Dimer Dynamics Reveals a Possible Allosteric Mechanism

**Figure 8:**
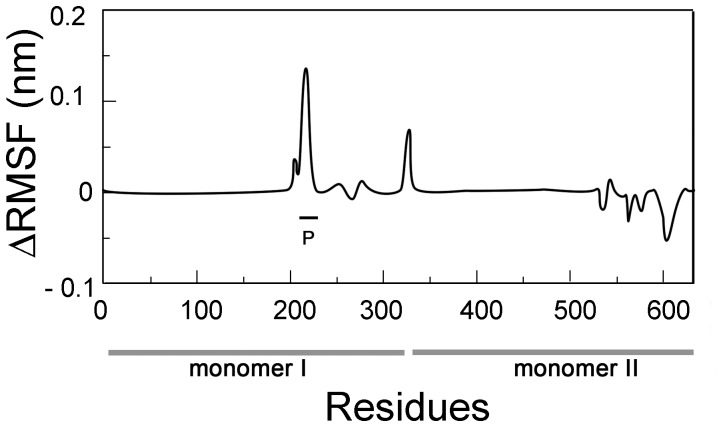
Effect of adenine nucleotide binding on PaNTD dimers. SBM simulations of PaNTD dimers bound to ATP or ADP were performed and the average fluctuation per residue (RMSF) was obtained. RMSF difference (ΔRMSF) between ADP and ATP bound dimers is shown. Monomer A: r. 1–329; monomer B: r. 330–658. P: Putative interaction site.

Molecular dynamics simulations allow us to explore the mechanistic details underlying allostery that are difficult to observe experimentally. For single domain proteins, such as Fdx [46], it has been corroborated that structured-based models are capable of capturing dynamical coupling between distal regions. We ran SBM simulations of PaNTD dimer bound to ADP or ATP at 0.95 of the melting temperature. It should be taken into account that in GHL proteins the N-terminal segment (L1) of one monomer is used to engage the ATPase site of the partner monomer. This region provides amino acids to directly co-ordinate bound nucleotide [15], [11]. This and other interaction between monomers are missing in the MD simulations performed in PaNTD monomer (see Figure 4) and included in these ones. When the difference in the Cα fluctuation (ΔRMSF) between ADP and ATP bound PaNTD dimer were calculated (Figure 8), an increase of up to 0.15 nm was observed in the putative interaction site detected with mix solvent MD (residues 208–225) of the ADP bound dimer. Thus, putative interaction site detected in mixed solvent MD (r. 209–230) is more flexible in ADP bound than in ATP bound PaNTD dimer. Differences observed in RMSF where not due to differences in the contact maps of the dimers, since the only differences present between both dimers were contacts of PaNTD with γ-phosphate in PaNTD-ATP complex.

These results suggest that the presence of ADP/ATP could act as a switch to couple/uncouple the motion of nucleotide binding site with a putative protein-protein site. It is interesting to note that smaller differences in RMSF values have been proved to be mechanistically relevant for Fdx [46].

### Influence of Nucleotide Binding on PaNTD-PaCTD Interaction

**Figure 9:**
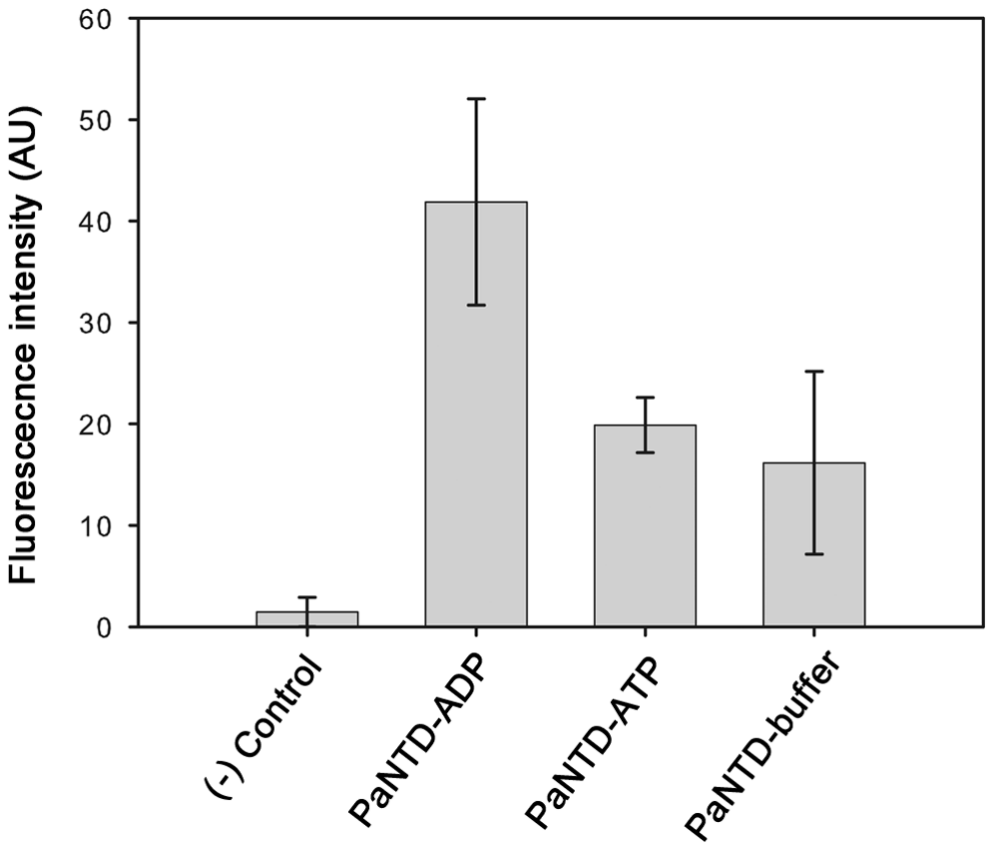
Analysis of PaNTD-PaCTD interaction using far Western assays. Purified PaCTD (6.5 pmol) and BSA (20 pmol) with no Histag were spotted onto nitrocellulose membranes. The membranes were incubated with His6-PaNTD (0.6 µM) with buffer B, buffer plus ADP 0.1 mM or ATP 0.1 mM followed by immunochemical detection of His6-PaNTD as described in Material and methods. The fluorescence intensity was measured using imageJ. Error bars represent the standard deviation from triplicate experiments. AU: Arbitrary units.

Since theoretical results of SBM MD and mixed-solvent MD indicate that PaNTD protein-protein interactions could be nucleotide modulated, we tested PaNTD-PaCTD binding capacity in absence or in presence of ATP or ADP by far western (Figure 9 and Figure S7). PaCTD spotted onto nitrocellulose membranes was incubated in buffer with His-tag PaNTD in absence or in presence of ATP or ADP, and PaCTD-PaNTD complexes were revealed and quantified using a His-tag antibody. Whereas no difference where observed in PaNTD-PaCTD binding when PaNTD was incubated with buffer or ATP, an increased interaction was observed when incubated with ADP (Figure 9). PaNTD-BSA binding was used as a negative control whereas His-PaNTD was directly spotted onto membranes as a positive control (Figure S7). Far western results were statistically analyzed using an analysis of variance (ANOVA) followed by a Tuckey HSD test. These tests allowed us to determine that mean NTD-CTD interaction in presence of ADP was significantly higher than NTD-CTD interaction in presence of ATP or in absence of nucleotide (buffer) with a p value of 0.05 and 0.01, respectively. Also, no differences in NTD-CTD interaction were found for ATP vs. buffer incubation.

These results are consistent with theoretical data mentioned before, and indicate that binding of adenosines nucleotides could differentially affect the exposure of protein-protein interaction sites. This is in agreement with the assignment of a main role of nucleotide binding in PaMutL endonuclease activity modulation [5], [7], [9].

Nevertheless, further experiments would reinforce this observation and help to elucidate adenine nucleotides role in NTD-CTD interaction.

## Discussion

Several MutL homologues from organisms lacking MutH have been shown to possess an endonuclease activity that requires the integrity of a metal-binding motif located in MutL C-terminal domain [3]–[6], [9], [10]. In human and yeast MutLα, endonuclease activity has been proved to be nick-directed and mismatch dependent [3], [4]. Knowing how this endonuclease activity is modulated becomes essential to understand the regulation of mismatch repair process in organisms lacking of MutH. MutL ATPase cycle has been demonstrated to regulate conformational transitions as well as enzymatic activity of MutL in Methyl-directed and MutH-less pathways [15], [40], [47]. Particularly, ATP binding is involved in the regulation of MutL endonuclease activity although its role is not fully understood yet [5], [7], [9]. *E. coli* MutL N-terminal domain LN40 (EcNTD) has been well characterized [12], [15]. Nevertheless, to better understand the regulation of MutL CTD endonuclease activity, a deep characterization of NTD from MutL homologues with endonuclease activity is needed.

*P. aeruginosa* MutL (PaMutL) endonuclease activity has recently been described, and ATP binding was found to inhibit it [9]. Also, due to the matchmaker role of MutL in the MMRS, the study of its different interaction interfaces and the role played by the nucleotide is of interest. In this work we have focused on the characterization of the interaction interfaces of PaMutL N-terminal domain (PaNTD), the role played by the nucleotide binding and also tried to gain insight into the NTD allosteric control of CTD endonuclease activity. Our experimental results indicate that PaNTD is capable of dimerization even in the absence of nucleotide, and that the addition of ADP or ATP further displaces the equilibrium toward the dimeric form. On the contrary, EcNTD only dimerizes when bound to the non-hydrolysable ATP analogue AMPPNP ([11] and this work). Structural analysis of the MD trajectories for EcNTD and PaNTD allowed us to comprehend the differential behavior observed in the oligomeric state of both proteins. All-atom MD simulations show that the α-helical structure of the ATP lid of EcNTD in the apo form is lost, while in the apo form of PaNTD, as well as in the holo form of both proteins, this structure is retained. This would indicate that the ATP lid, which is an important segment of the dimerization interface of the NTD, is intrinsically more stable in PaNTD than in the homologue EcNTD.

Neither human PMS2 nor yeast ScPMS1 MutL homologue can form homodimers. In the monomeric hPMS2 NTD crystal structure the ATP lid residues are disordered, even when AMPPNP is bound [13] (PDB:1H7U). The same is observed in the ATP-bound monomeric human Mlh1 NTD structure (PDB: 3NA3) where ATP lid residues do not diffract and are therefore not observed in the crystal. Therefore, ATP lid could be expected only to be fully order in the heterodimer hMutlα (hPMS2/hMlh1). In the also monomeric yeast PMS1 NTD, that crystallizes with two AMPPNP bound molecules in the asymmetric unit, ATP lid residues are disordered in molecule A, while they are fully order in molecule B (PDB: 3H4L) [14]. This indicates that PMS1 ATP lid could be expected only to be fully order in the heterodimer. This is in agreement with the fact that no differences in RMSF values were shown in SBM MD simulations when apo, ATP and ADP-bound form were compared. Taking everything into account, it is tempting to propose ATP lid ordering as an event related to nucleotide binding but not necessarily concomitant, but indispensable for NTD dimerization.

Although secondary structure is maintained, apo PaNTD SBM simulations show an increased mobility in ATP lid residues, in agreement with results with mix solvent MD which evidence density loss around it. RMSF values obtained from MD simulations using structure based models indicate that dimerization interface APO and ADP bound EcNTD would be similar and differed from the ATP bound. This is in agreement with the fact that EcNTD dimerizes only in presence of ATP [15]. On the other hand, ADP and ATP bound PaNTD dimerization interface behave similarly and differ from the APO state, which reflects on PaNTD dimerization in presence of either ADP or ATP. SBM MD simulations gave a more complete picture of the differences showing that the whole dimerization interface of EcNTD is destabilized when the ATP is removed or replaced by ADP (Figure 4). On the other hand, the conformations sampled by PaNTD are the same regardless of the type of nucleotide bound. The structure of PaNTD is destabilized only in the apo form, but in this case the conformational fluctuations observed in the apo PaNTD are smaller than in the case of EcNTD. A similar behavior to the observed for PaNTD dimerization interface was found for *Bacillus subtilis* and *Termotoga maritima* MutL NTD (BsNTD and TmNTD, respectively). SBM simulations are in agreement with experimental results that indicate that TmMutL forms a full dimer in presence of ADP.

PaNTD ATP lid possesses a higher α-helix propensity, as determined using the AGADIR predictor. *Thermotoga maritima* MutL (TmMutL) forms a full dimer in presence of ADP [48]. TmMutL ATP lid also has an increased predicted content of α-helix. These changes in ATP lid sequences could contribute to the differences with EcNTD observed for PaNTD, and TmMutL [48]. It is interesting to note that the secondary structure prediction algorithm AGADIR predicts the same order in stability of the helix in the ATP lid for the two proteins.

The simulations in mixed solvent, as expected, signal the dimerization interface of PaNTD as a region prone to be desolvated in the holo form. In the apo form this mark is less intense, in line with the observed lower tendency to dimerize with respect to the holo forms. Simulations in mixed solvent also signal two other regions of PaNTD which are prone to be desolvated. By comparison with the well caracterized EcNTD, one of these regions can be assigned to a protein-DNA interaction patch. This region poses a positive electrostatic potential which is conserved among the MutL family [15]. Particularly, the important EcNTD residue Arg-266 involved in DNA interaction is conserved in PaNTD [15]. The server DNABindR [44] for prediction of protein-DNA interaction sites was used to predict PaNTD residues involved in DNA contact. We observed a correspondence between these residues and the ones spotted out in mixed solvent MD. These simulations also evidenced a loss in iPrOH density in the DNA binding patch for nucleotide free PaNTD. This may indicate that MutL DNA binding is enhanced by nucleotide binding. However AMPPNP but not ATP enhaces the interaction between EcMutL and ssDNA, probably because of the dimerization of the N-terminal [15]. On the other hand, in PMS2 N-terminal domain, that does not form a homodimer upon association with ATP, DNA binding is not affected by the presence of ATP [13]. These results may indicate dimerization rather than nucleotide binding to be involved in the modulation MutL-DNA interaction, at least for these two homologues. This, however, does not rule out the posibility of nucleotide binding to regulate paMutL-DNA interaction.

The other region signaled in mixed solvent simulations has no assigned function and we postulate it to be a putative protein-protein interaction interface. This interaction interface is located opposite to the dimerization face, encompassing residues 209–230 and 245–252. Deuterium incorporation assays have previously allowed the detection of a significant ATP-dependent structural rearrangement in the homologue region of the *Aquifex aeolicus* NTD and that this region may be required for the direct interaction between the NTD and CTD [17]. SBM simulations of PaNTD dimers bound to ADP or ATP indicate that adenine nucleotide binding site is communicated with this putative interaction patch located in residues 208–230. Thus, nucleotide binding could differentially modulate protein-protein interactions of PaNTD. This is also consistent with our experimental results that indicate that PaMutL NTD-CTD interaction is enhanced in the presence of ADP, but not in the presence of ATP. The modulation of such interaction could be significant for PaMutL activity.

Since ATP has been proved to inhibit PaMutL endonuclease activity [9], and ADP but not ATP enhanced NTD-CTD interaction, it is tempting to infer a fully dimerized MutL ADP-bound complex capable of DNA nicking. For PaMutL, one can hypothesize that while ATP-bound PaMutL can load to the DNA strand, it is not allowed to cut. ATP hydrolysis and generation of ADP bound PaMutL would still be able to remain loaded to DNA, and nick the newly synthesize strand. Further studies could give experimental support to this model.

### Conclusions

Results obtained in this study provide insight into how *P. aeruginosa* MutL activity could be modulated and allow inferring the mechanistic differences that may arise among Mismatch Repair System functioning in organism with MutL homologues that carry or not endonuclease activity.

## Supporting Information

Figure S1 **PaNTD and EcNTD all atom MD evaluation.** (A) Time evolution of RMSD values for apo and ATP bound PaNTD and EcNTD. Only Cα atoms were taken into account. The first 12 and 9 residues of PaNTD and EcNTD, respectively, were not included. (B) The difference in total secondary structure content between the apo and the holo forms of PaNTD (black) and EcNTD (red) along the MD was calculated using do_dssp. The number of residues within secondary structures was determined.
(TIF)

Figure S2 **Time evolution of dimerization interface secondary structure.** Dimerization interface secondary structure along the 200 ns of the MD simulation was determined for PaNTD and EcNTD, with or without ATP, using DSSP. A) ATP lid (PaNTD r. 78–101, EcNTD 74–97); B) L1 (PaNTD r. 6–24, EcNTD r. 2–20); C) L2 (PaNTD r. 154–166, EcNTD r. 150–162); D) L3 (PaNTD r. 302–316, EcNTD 299–313) and E) L45 (PaNTD r. 130–135, EcNTD 126–131).
(TIF)

Figure S3 **Percentage of predicted helical content of EcNTD, PaNTD and TmNTD ATP lid using AGADIR.** The sequence of the helical region of EcNTD (r. 79–90), PaNTD (r. 83–94) and Termotoga maritima NTD (TmNTD, 85–96) ATP lid were used to calculate the tendency of these peptides to form alpha helix.
(TIF)

Figure S4 **PaNTD oligomeric state after ON incubation in EDTA.** PaNTD (10 µM) in 20 mM HEPES (pH: 7.4), 150 mM KCl, 10% glycerol, 5 mM MgCl_2_ and 1 mM DTT and in absence of added nucleotides was incubated ON without (lane 2) or with 1 mM EDTA (lane 3). The oligomeric state of incubated proteins was determined by native PAGE in order to determine if PaNTD dimers were resistant to EDTA incubation. BSA was used as a molecular weight marker (lane 1).
(TIF)

Figure S5 **Oligomeric state of EcNTD.** Crosslinking analysis (DSS 2.5 mM) of the EcNTD oligomeric state incubated ON in absence (EDTA incubated) or in presence of ADP, ATP or AMPPNP (lanes 3–6) were performed. Lane 1: molecular weight markers; Lane 2: control with no DSS.
(TIF)

Figure S6 **paNTD putative DNA binding site.** DNAbindR [44] server was used to predict PaNTD residues involved in DNA binding. (A) The alignment of Subdomain II (SBII) of PaNTD and EcNTD sequences is shown, along with the residues predicted with DNAbindR to be involved in DNA contact (#). Secondary structure of EcNTD residues involved in binding are represented. The nomenclature used corresponds to the one used in LN40 crystal structure (PDB: 1B63). PASBII: PaNTD sub-domain II sequence. ECSBII: EcNTD sub-domain II sequence. The predicted PaNTD DNA binding residues are shown in red in the 3D model of the PaNTD dimer from a front (B) and bottom view (C). The figure was generated using VMD [39].
(TIF)

Figure S7 **Analysis of PaNTD-PaCTD interaction using far Western assays.** Purified PaCTD with no-tag (1) BSA (negative control) (2) andHis6-PaNTD (20 pmol) as a positive control (3) were spotted onto nitrocellulose membranes. The membranes were incubated with His6-PaNTD with buffer, buffer plus ADP 0.1 mM or ATP 0.1 mM followed by immunochemical detection of His6-PaNTD as described in Material and methods.
(TIF)
