# Supplementary figures and images for: Analysis of the Interaction Interfaces of the N-Terminal Domain from Pseudomonas aeruginosa MutL
Source: PLoS One. 2013 Jul 26;8(7):e69907. doi: 10.1371/journal.pone.0069907 (PMC3724809; doi:10.1371/journal.pone.0069907)

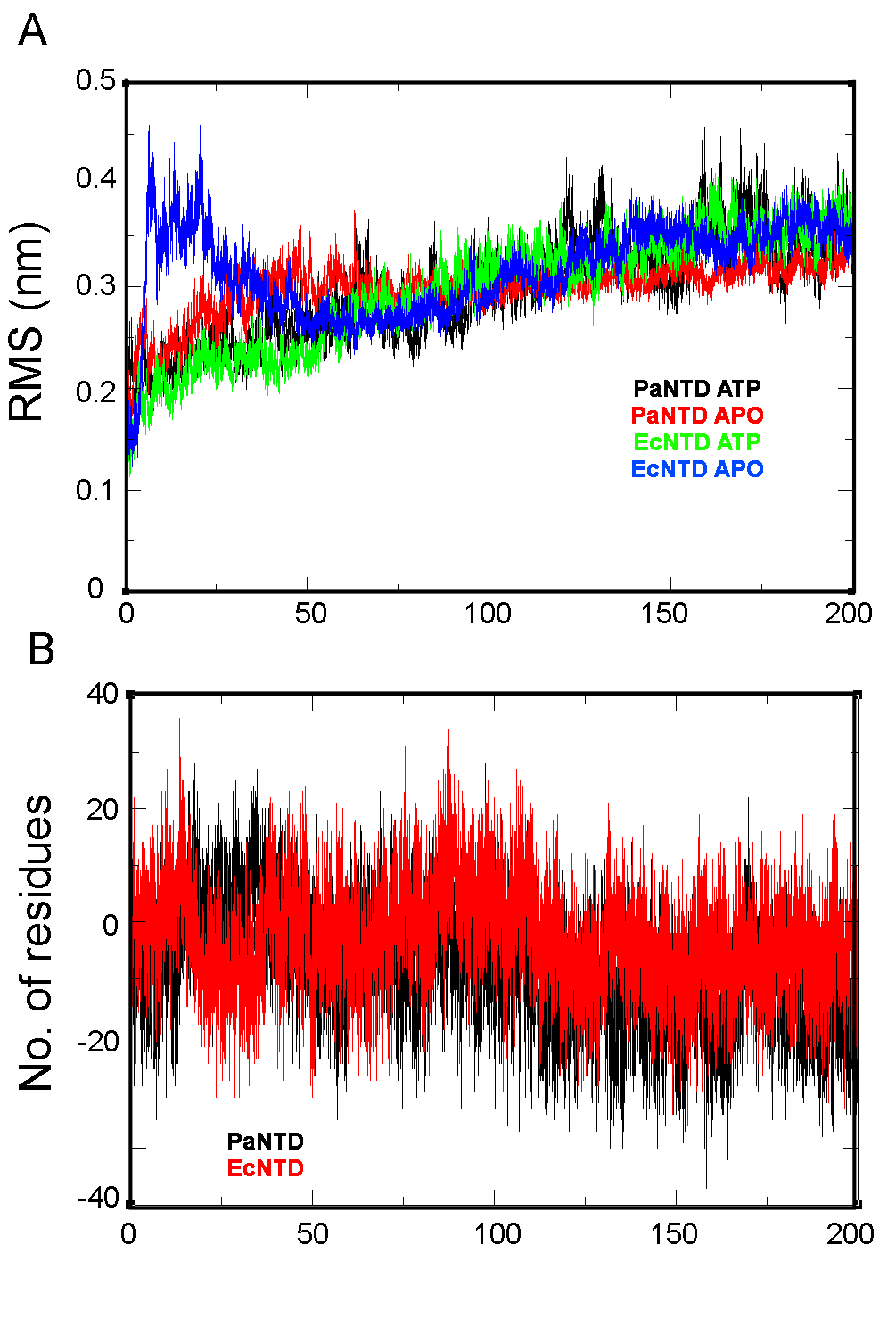

Supplement: Figure S1 — PaNTD and EcNTD all atom MD evaluation. (A) Time evolution of RMSD values for apo and ATP bound PaNTD and EcNTD. Only Cα atoms were taken into account. The first 12 and 9 residues of PaNTD and EcNTD, respectively, were not included. (B) The difference in total secondary structure content between the apo and the holo forms of PaNTD (black) and EcNTD (red) along the MD was calculated using do_dssp. The number of residues within secondary structures was determined. (TIF) [file pone.0069907.s001.tif]

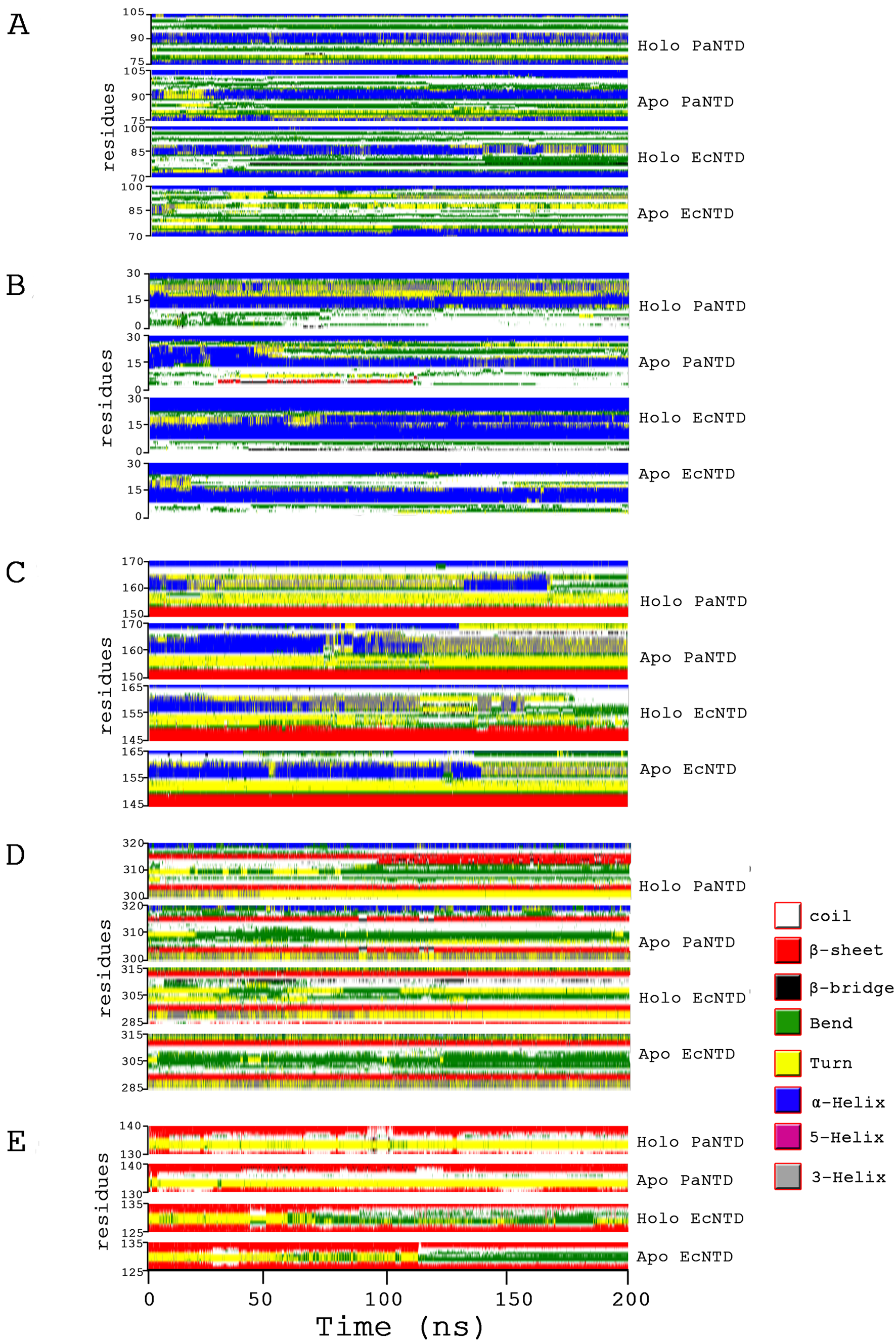

Supplement: Figure S2 — Time evolution of dimerization interface secondary structure. Dimerization interface secondary structure along the 200 ns of the MD simulation was determined for PaNTD and EcNTD, with or without ATP, using DSSP. A) ATP lid (PaNTD r. 78–101, EcNTD 74–97); B) L1 (PaNTD r. 6–24, EcNTD r. 2–20); C) L2 (PaNTD r. 154–166, EcNTD r. 150–162); D) L3 (PaNTD r. 302–316, EcNTD 299–313) and E) L45 (PaNTD r. 130–135, EcNTD 126–131). (TIF) [file pone.0069907.s002.tif]

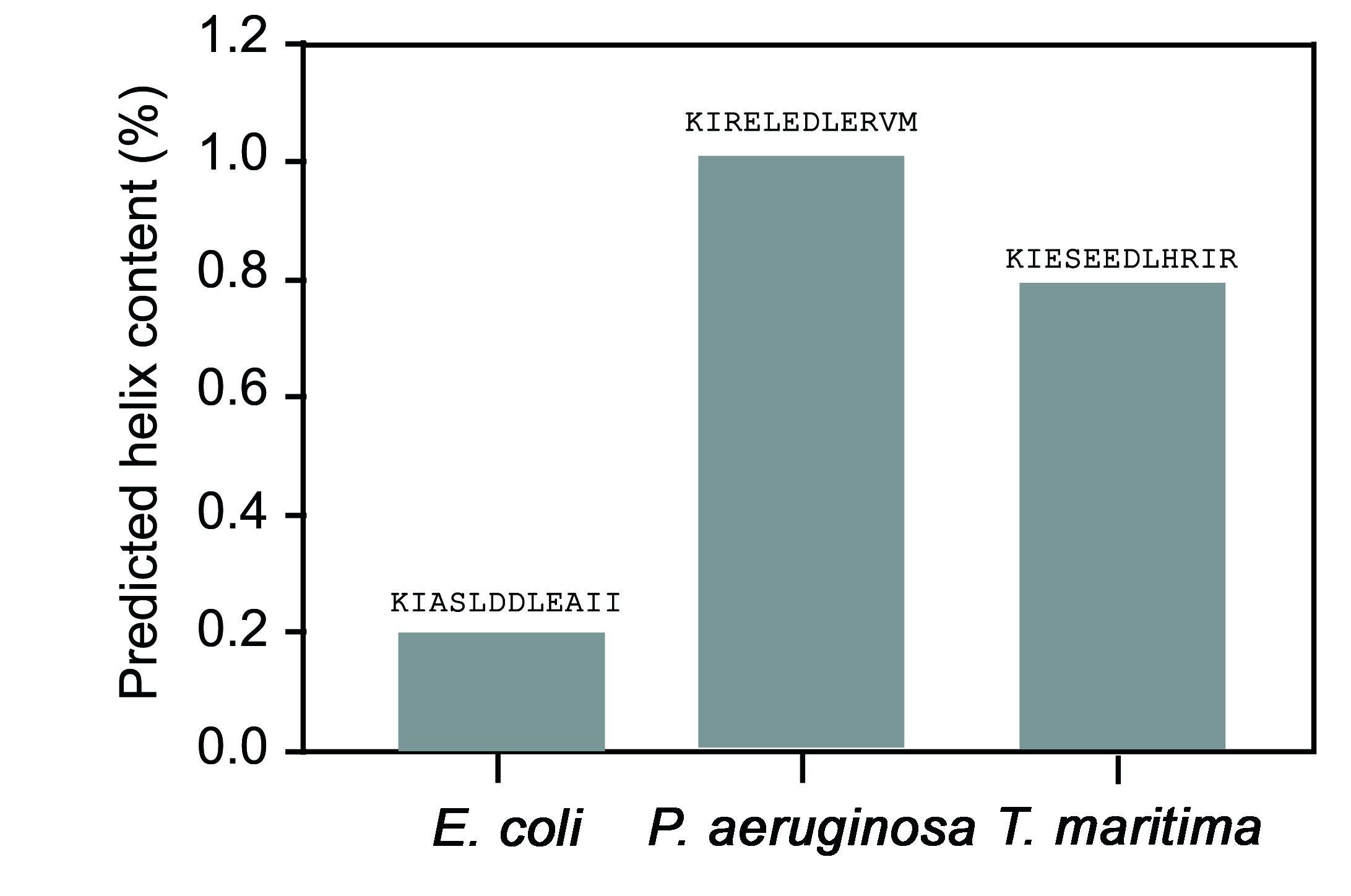

Supplement: Figure S3 — Percentage of predicted helical content of EcNTD, PaNTD and TmNTD ATP lid using AGADIR. The sequence of the helical region of EcNTD (r. 79–90), PaNTD (r. 83–94) and Termotoga maritima NTD (TmNTD, 85–96) ATP lid were used to calculate the tendency of these peptides to form alpha helix. (TIF) [file pone.0069907.s003.tif]

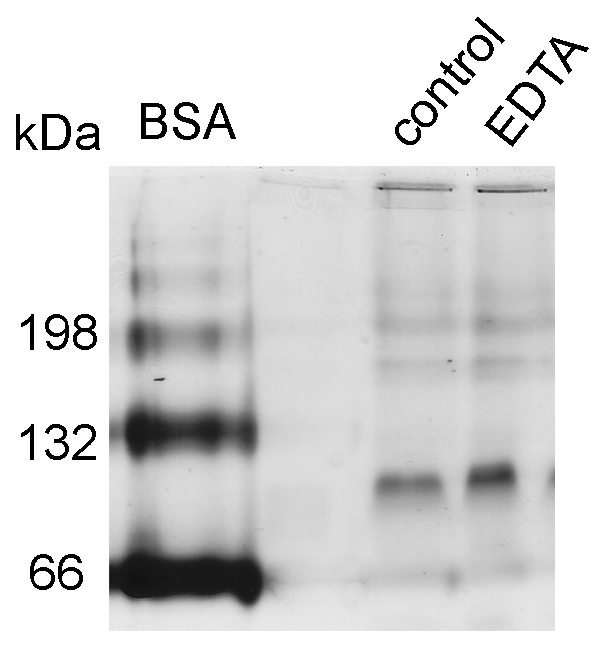

Supplement: Figure S4 — PaNTD oligomeric state after ON incubation in EDTA. PaNTD (10 µM) in 20 mM HEPES (pH: 7.4), 150 mM KCl, 10% glycerol, 5 mM MgCl2 and 1 mM DTT and in absence of added nucleotides was incubated ON without (lane 2) or with 1 mM EDTA (lane 3). The oligomeric state of incubated proteins was determined by native PAGE in order to determine if PaNTD dimers were resistant to EDTA incubation. BSA was used as a molecular weight marker (lane 1). (TIF) [file pone.0069907.s004.tif]

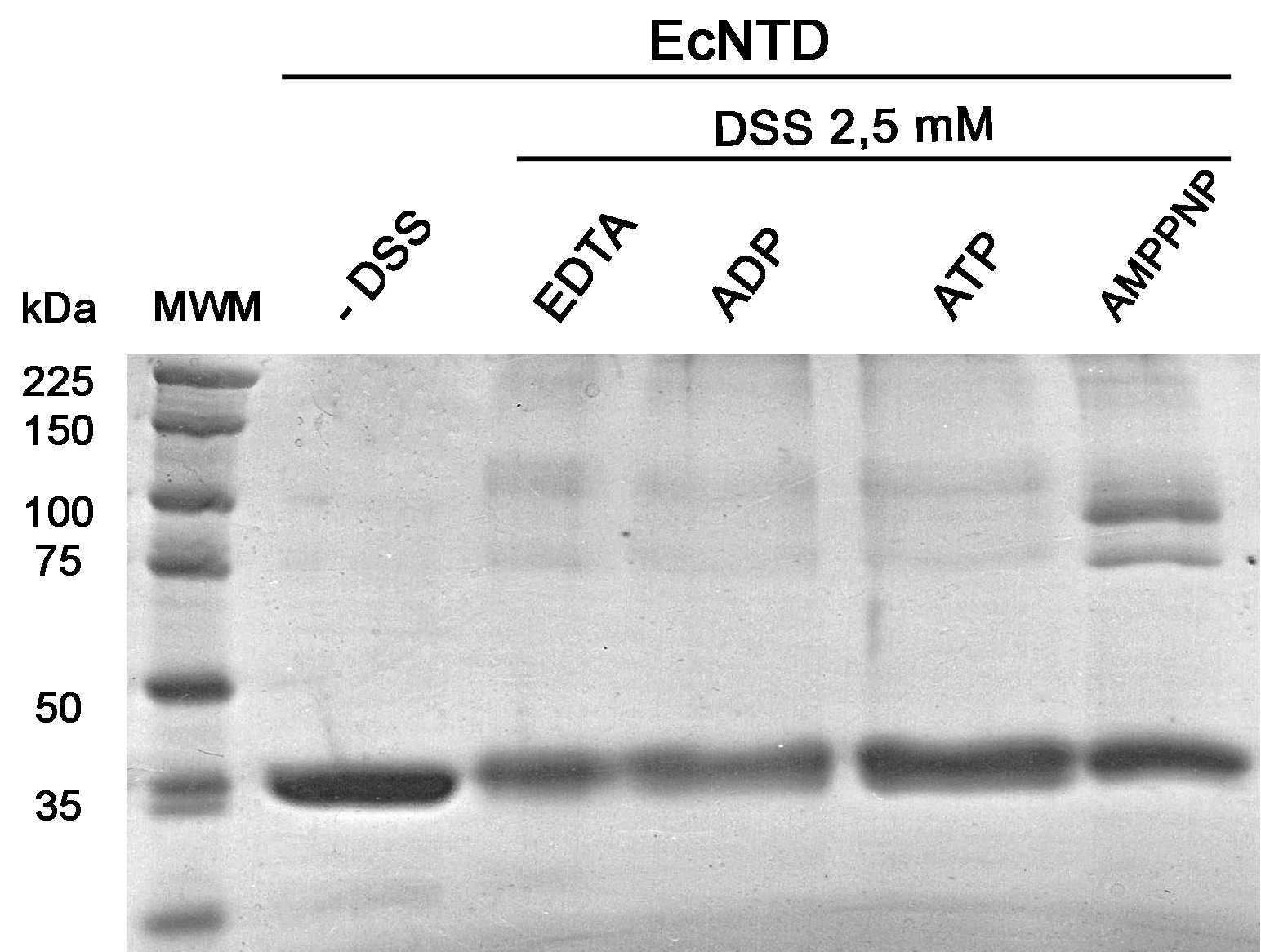

Supplement: Figure S5 — Oligomeric state of EcNTD. Crosslinking analysis (DSS 2.5 mM) of the EcNTD oligomeric state incubated ON in absence (EDTA incubated) or in presence of ADP, ATP or AMPPNP (lanes 3–6) were performed. Lane 1: molecular weight markers; Lane 2: control with no DSS. (TIF) [file pone.0069907.s005.tif]

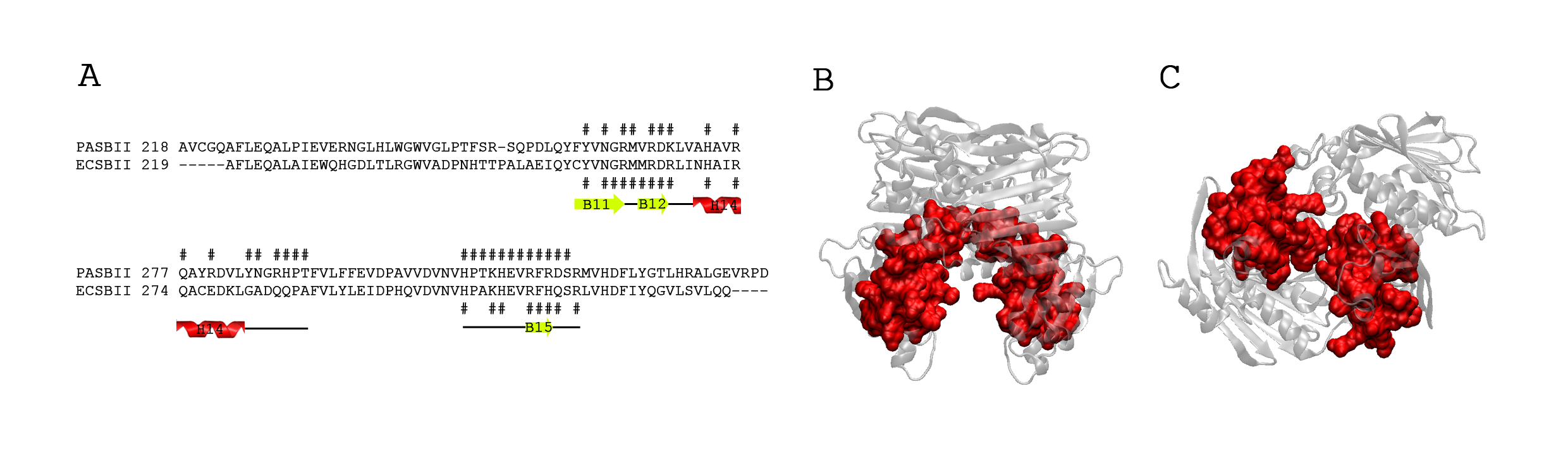

Supplement: Figure S6 — paNTD putative DNA binding site. DNAbindR [44] server was used to predict PaNTD residues involved in DNA binding. (A) The alignment of Subdomain II (SBII) of PaNTD and EcNTD sequences is shown, along with the residues predicted with DNAbindR to be involved in DNA contact (#). Secondary structure of EcNTD residues involved in binding are represented. The nomenclature used corresponds to the one used in LN40 crystal structure (PDB: 1B63). PASBII: PaNTD sub-domain II sequence. ECSBII: EcNTD sub-domain II sequence. The predicted PaNTD DNA binding residues are shown in red in the 3D model of the PaNTD dimer from a front (B) and bottom view (C). The figure was generated using VMD [39]. (TIF) [file pone.0069907.s006.tif]

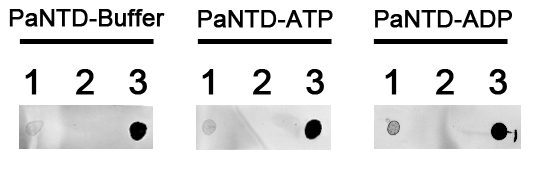

Supplement: Figure S7 — Analysis of PaNTD-PaCTD interaction using far Western assays. Purified PaCTD with no-tag (1) BSA (negative control) (2) andHis6-PaNTD (20 pmol) as a positive control (3) were spotted onto nitrocellulose membranes. The membranes were incubated with His6-PaNTD with buffer, buffer plus ADP 0.1 mM or ATP 0.1 mM followed by immunochemical detection of His6-PaNTD as described in Material and methods. (TIF) [file pone.0069907.s007.tif]
